# An innovative tool for moving malaria PCR detection of parasite reservoir into the field

**DOI:** 10.1186/1475-2875-12-405

**Published:** 2013-11-09

**Authors:** Lydie Canier, Nimol Khim, Saorin Kim, Vincent Sluydts, Somony Heng, Dany Dourng, Rotha Eam, Sophy Chy, Chanra Khean, Kaknika Loch, Malen Ken, Hokkean Lim, Sovannaroath Siv, Sochantha Tho, Pascal Masse-Navette, Charlotte Gryseels, Sambunny Uk, Karel Van Roey, Koen Peeters Grietens, Mao Sokny, Boukheng Thavrin, Char Meng Chuor, Vincent Deubel, Lies Durnez, Marc Coosemans, Didier Ménard

**Affiliations:** 1 Institut Pasteur du Cambodge, Phnom Penh, Cambodia; 2 Institute of Tropical Medicine, Antwerp, Belgium; 3 National Center for Parasitology, Entomology and Malaria Control, Phnom Penh, Cambodia; 4 Department of Biomedical Sciences, University of Antwerp, Antwerp, Belgium

## Abstract

**Background:**

To achieve the goal of malaria elimination in low transmission areas such as in Cambodia, new, inexpensive, high-throughput diagnostic tools for identifying very low parasite densities in asymptomatic carriers are required. This will enable a switch from passive to active malaria case detection in the field.

**Methods:**

DNA extraction and real-time PCR assays were implemented in an “in-house” designed mobile laboratory allowing implementation of a robust, sensitive and rapid malaria diagnostic strategy in the field. This tool was employed in a survey organized in the context of the MalaResT project (NCT01663831).

**Results:**

The real-time PCR screening and species identification assays were performed in the mobile laboratory between October and November 2012, in Rattanakiri Province, to screen approximately 5,000 individuals in less than four weeks and treat parasite carriers within 24–48 hours after sample collection. An average of 240 clinical samples (and 40 quality control samples) was tested every day, six/seven days per week. Some 97.7% of the results were available <24 hours after the collection. A total of 4.9% were positive for malaria. *Plasmodium vivax* was present in 61.1% of the positive samples, *Plasmodium falciparum* in 45.9%, *Plasmodium malariae* in 7.0% and *Plasmodium ovale* in 2.0%.

**Conclusions:**

The operational success of this diagnostic set-up proved that molecular testing and subsequent treatment is logistically achievable in field settings. This will allow the detection of clusters of asymptomatic carriers and to provide useful epidemiological information. Fast results will be of great help for staff in the field to track and treat asymptomatic parasitaemic cases. The concept of the mobile laboratory could be extended to other countries for the molecular detection of malaria or other pathogens, or to culture vivax parasites, which does not support long-time delay between sample collection and culture.

## Background

In Southeast Asia, the incidence of malaria (mainly *Plasmodium falciparum*) has significantly decreased in the past ten years [1,2]. However, the emergence of artemisinin resistance in *P. falciparum*, firstly detected in 2008–2009 in western Cambodia [3-5] and confirmed in Thailand [6], Myanmar [7] and Vietnam [8], threatens these efforts.

In very low transmission settings such as Cambodia, asymptomatic infections remain the major reservoir of malaria parasites contributing to maintain disease transmission [9-11]. As a consequence, the detection and treatment of the asymptomatic carriers is a crucial step in progress towards malaria elimination [12]. This represents a new challenge as the proportion of asymptomatic parasite carriers is unknown. Moreover, standard diagnostic tools such as the rapid diagnostic test (RDT) or microscopy examination of a blood slide often fail to detect low parasitaemia [13,14]. To achieve the goal of malaria elimination in areas with low transmission intensity, and especially in Cambodia where artemisinin-based combination therapy (ACT) efficacy is declining [15], the diagnostic toolbox needs to be enriched with inexpensive diagnostics capable of identifying extremely low parasite densities in asymptomatic individuals in the field. The information acquired from such tools will enable determination of the areas of residual malaria transmission in the country and will guide new parasite and vector control strategies required to re-orientate national control programmes towards the achievement of elimination [16].

Usually, routine diagnostics in the field are performed by microscopy and/or RDT. Good RDT and well-performed microscopy can detect parasitaemia of at least 50–200 parasites/μl [17], which is sufficient for the management of symptomatic cases, but less informative in detecting asymptomatic cases [13]. Moreover, microscopy remains labour intensive and time consuming. Its quality in field conditions is often inadequate and limited by factors such as the quality of reagents, microscopist skill and poorly maintained equipment. On the other hand, RDT, which is simple to use, has several drawbacks, such as a variable detection threshold or field stability [18]. For more than a decade, the performance of malaria diagnostic tests has been considerably improved with the introduction of molecular assays [19]. Several PCR-based assays detecting parasite nucleic acids have been described so far, but surprisingly, microscopy is still recognized as the gold standard for laboratory confirmation. The nested PCR targeting the *small subunit ribosomal RNA 18S* (*18S rRNA*) gene, described by Snounou *et al*. in 1993 [19] and improved in 1999 [20], remains the most commonly used method. Although PCR methods show lower detection threshold compared to microscopy [17,21], nested PCR assays are time consuming, very prone to contamination and not suited for high-throughput testing. The real-time PCR approach has the potential to overcome these limitations, offering a simple, time-effective and even more sensitive diagnostic option [22]. Most of the PCR-based assays target the *18S rRNA* gene [23,24], whereas detecting mitochondrial genes such as *cytochrome b* gene has been suggested to be a more sensitive approach due to their higher copy numbers in the parasite genome (20–100 copies) [11,25-27].

In the current study, a strategy based on real-time PCR detection of the *Plasmodium cytochrome b* gene has been developed. The rationale was to screen as a first step samples for malaria parasites using genus-specific primers (named “real-time PCR screening”), allowing treatment of positive cases in <24-48 hours in field settings. In a second step, a PCR assay capable of identifying the malaria species was carried out on positive samples. This assay was designed as a nested real-time PCR with four separate reactions (*P. falciparum*, *Plasmodium vivax*, *Plasmodium malariae* and *Plasmodium ovale*) (named “nested real-time PCR species”). Multiplexing assay was avoided to increase the sensitivity for detecting minor species in mixed infections.

Adaptation of the assay to large-scale epidemiologic studies required the implementation of a rapid DNA extraction technique. Blood samples were collected in the field on filter paper in a 96-well plates format, and DNA was extracted using a simple and fast chelex-boiling method described previously [12]. The molecular detection method described here was optimized and validated using in-house controls, and compared to the *18S rRNA* nested PCR on a set of DNA samples. A quality assurance system was developed to monitor the performance of both DNA extraction and PCR over time.

Currently, malaria molecular diagnosis using PCR-based method has been restricted to well-equipped laboratories. As a consequence, this hampers its use for active malaria case detection because the timely feedback of results does not allow the treatment of identified cases [12]. In the present study, DNA extraction and PCR technologies were implemented in an “in-house” designed mobile laboratory allowing a robust, sensitive and rapid malaria diagnostic strategy into the field. This innovative approach has been employed in the context of a two-year study “Repellents as added control measure to long-lasting insecticidal nets to target the residual transmission in Southeast Asia: a step forward to malaria elimination” (MalaResT project, NCT01663831).

## Methods

### Mobile laboratory

**Figure 1:**
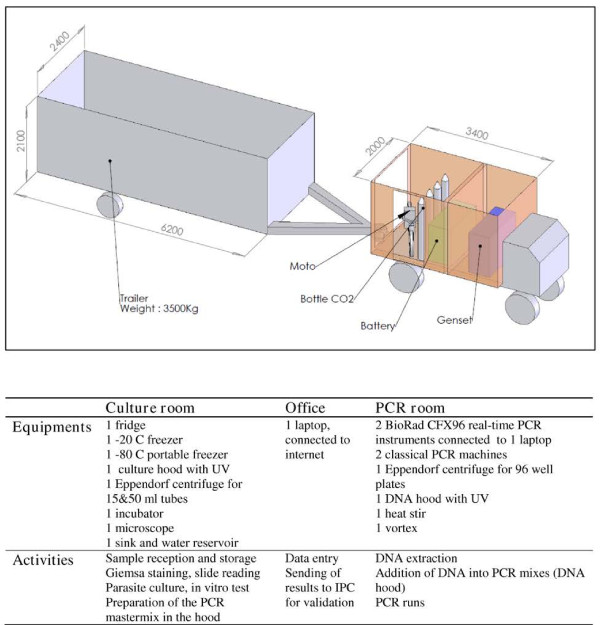
Design of the mobile laboratory.

The mobile laboratory was designed in November 2011, manufactured by FEMIL [28] in January-April 2012 and available in Phnom Penh in August 2012. The map of the mobile laboratory, pictures, equipment and activities are presented in Figure 1 and Additional file 1. The mobile laboratory has a total surface of approximately 15 sq m, including three air-conditioned rooms (PCR room, office and culture room) fully equipped to perform DNA extraction, real-time PCR assays, microscopy and parasite culture in a completely autonomous way.

The mobile laboratory is towed by a truck equipped with a 15 KVA generator, eight batteries, a cabin for three passengers, and enough storage space for equipment and consumables. The total length of the convoy is ~13 m. A system of cylinders maintains a stable horizontal orientation.

During day activities, equipment can work with local electricity supply when available, or with the generator that automatically starts when the power is interrupted. Sensitive devices such as real-time PCR equipment are connected to a 3KVA UPS to overcome the transition of electricity supply and irregular voltage. During the night or during transport, the batteries ensure the electricity supply of the fridge, freezers, incubator, lights and ventilation (3,000 watts, capacity up to ten hours back-up).

### Real-time PCR validation

#### Real-time PCR screening

*Plasmodium falciparum* ring-stage parasites obtained from synchronized reference 3D7 parasites cultures were used as positive controls.

#### Optimization

For the optimization and evaluation of the performance of the real-time PCR screening, 200 μl red blood cell pellet (~3% parasitaemia) were extracted with the QIAamp DNA Blood Mini kit® (Qiagen, Courtaboeuf, France). DNA was eluted with 200 μl of water and DNA concentration was measured with a spectrophotometer (Nanodrop 2000®, Wilmington, USA) and adjusted at 1 ng/μl. Ten-fold dilutions were prepared from 10^-1^ ng/μl to 10^-6^ ng/μl with DNAse/RNAse-free water.

After optimization, two concentrations (10^-1^ and 10^-3^ ng/μl) were selected as positive controls. In parallel, 200 μl of uninfected blood were extracted by same method, adjusted at 10^-1^ ng/μl and used as negative control.

#### Threshold of detection

To assess the detection threshold of the entire process (including DNA extraction and volume of blood analysed), cultured 3D7 ring-stage parasites were adjusted to approximately 1% parasitaemia and serially diluted ten-fold using uninfected blood from the blood bank, from 1,000 to 0.01 parasites/μl. Twenty μl of each dilution were spotted onto Whatman 3MM filter paper and air dried. Punches of 4-mm diameter were prepared and stored at -20°C. Blood spot confetti of uninfected blood were also prepared to serve as negative controls. DNA was extracted with Instagene® Matrix resin (ref. 732–6030, Bio-Rad, Singapore). Two concentrations (high positive control (HPC) at 500 parasites/μl and low positive control (LPC) at 5 parasites/μl) were selected and used as positive control in further analysis.

#### Real-time PCR species

For the validation of the four species-specific PCR assays, 400 bp fragment of the first round of amplification were generated and used to prepare *P. falciparum*, *P. vivax*, *P. malariae* and *P. ovale* plasmids. Plasmid DNA concentrations were assessed with a spectrophotometer (Nanodrop 2000®, Wilmington, USA). A ten-fold dilution series of each plasmid (from 10^-1^ ng/μl to 10^-9^ ng/μl) was prepared to evaluate the efficiency of each species-specific PCR assays.

### Method comparison

The real-time PCR screening and species identification assays were compared with the *18S rRNA* nested PCR described by Snounou *et al*. [24] on a cohort of 175 external DNA samples provided by FIND [29]. These samples were collected in 2012 in Uganda and Burkina Faso, and were previously characterised using the *18S rRNA* nested PCR. Upon reception at IPC, all samples were blindly re-tested with the genus-specific *18S rRNA* nested PCR (rPLU1 and rPLU 5 primers). Then, samples were screened for malaria using the real-time PCR screening assay, and positive samples were analysed for identification of malaria species using the real-time PCR species assay.

### High-throughput malaria PCR detection flow

**Figure 2:**
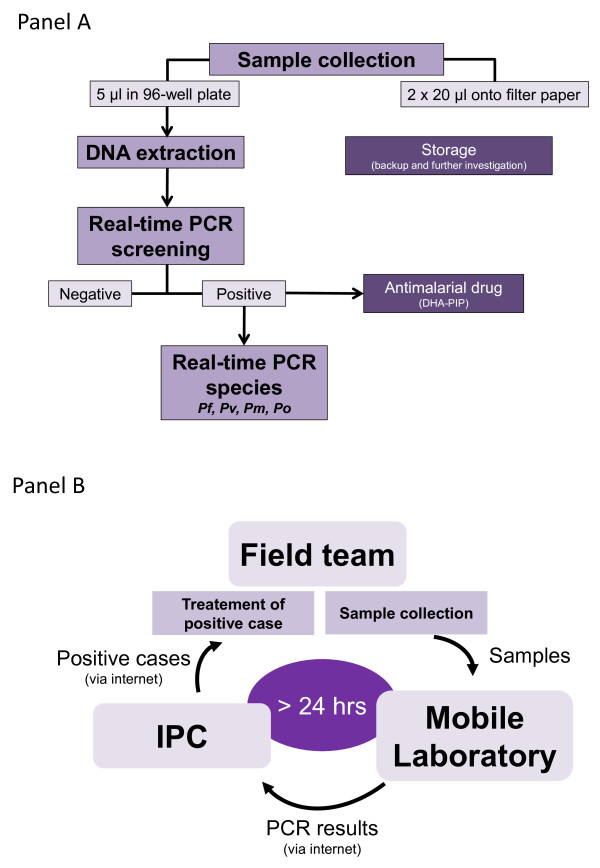
**The overview of the process used for high**-**throughput malaria PCR detection in the field (Panel A), including samples and data management (Panel B).**

An overview of the entire PCR detection process is provided in Figure 2 (Panel A).

### Sample collection

Blood samples from each participating individuals were collected in the field on filter paper from a finger prick in two different formats: 5 μl in a 96-well plate containing a 4-mm diameter Whatman 3MM filter paper using a capillary tube, and two spots of 20 μl on a standard Whatman 3MM filter paper (Additional file 2). Blood from the 96-well plates was used for malaria screening by real-time PCR, while 20-μl blood spots were air-dried, wrapped in aluminium foil, and stored at -20°C (used as back-up or for further analyses).

### DNA extraction

Blood spots were lysed overnight on 96-well plates at 4°C with 150 μl per well of HBS 1X/Saponin 0.5%. Sample were then washed twice with HBS 1X and DNA was extracted with Instagene® Matrix resin (Bio-Rad, Singapore) according to manufacturer’s instructions. An additional centrifugation step (4,000 rpm, 20 min) was added to limit the presence of inhibitors and a final volume of 50 μl of the supernatant was transferred into a new 96-well plate.

### Real-time PCR

Molecular detection and identification of *Plasmodium* parasites were performed in two steps: *Plasmodium* parasites were first detected by a “screening real-time PCR” with genus-specific primers targeting the *Plasmodium cytochrome b gene*. Secondly, DNA samples identified as positive for *Plasmodium* were analysed for malaria species using a nested real-time PCR assay. The first round of amplification was performed with genus-specific primers in a standard thermocycler. PCR products were then diluted 1:10 and analysed in four real-time PCR assays with primers targeting the same gene fragment and specifics to each species (*P. falciparum*, *P. vivax*, *P. malariae*, and *P. ovale*). All real-time PCR assays were followed by a melt curve analysis. Details are provided in Table 1.

**Table 1:** **Primers sequences and real**-**time PCR conditions used to detect *Plasmodium *** **species**

**Real-time PCR name**		**Primer name**	**Sequence (5′-3′)**	**Master mix**	**Assay parameters**	**Melt parameters**	**T° melt peak**
**Real-time PCR screening**		RTPCRScreening2_F	TGGAGTGGATGGTGTTTTAGA	Hot FirePol EvaGreen qPCR Mix Solis Biodyne 1X (#08-24-00020), Primers 150 nM, 5 μl DNA template, Total volume 20 μl	95°C-15 min 45 cycles: 95°C-15 sec/60°C-20 sec/72°C-20 sec 95°C-2 min 68°C-2 min	From 68 to 90°C, increment 0.2°C for 0.05 sec	76.4-78.4°C
		RTPCRScreening2_R	TTGCACCCCAATARCTCATTT				
**Nested real-time PCR species**	Primary PCR	RTPCRScreening2_F	TGGAGTGGATGGTGTTTTAGA	Hot FirePol DNA Pol. Solis BioDyne 1.25 U (#01-02-01000), dNTP 200 μM, MgCl2 2.5 mM, Primers 250 nM, 5 μl DNA template. Total volume 20 μl.	94°C -15 min 20 cycles: 94°C-30 sec/ 58°C -1 min/ 72°C-1 min 72°C-10 min	N/A	N/A (PCR product size: 400 bp)
		RTPCRSreening3_R	ACCCTAAAGGATTTGTGCTACC				
	Nested real-time PCR *Pf*	Pf_RTPCR_F	ATGGATATCTGGATTGATTTTATTTATGA	Hot FirePol EvaGreen HRM Mix Solis Biodyne 1X (#08-33-00001), Primers 250 nM, 5 μl template Primary PCR products 1:10, Total volume 20 μl	95°C-15 min 35–45 cycles*: 95°C-10 sec/62°C-20 sec/72°C-25 sec 95°C-1 min 40°C-1 min	From 65 to 90°C, increment 0.2°C for 0.05 sec.	78.6-79.6°C
		Pf_RTPCR_R	TCCTCCACATATCCAAATTACTGC				
	Nested Real-time PCR *Pv*	Pv_RTPCR_F	TGCTACAGGTGCATCTCTTGTATTC				74.8-75.8°C
		Pv_RTPCR_R	ATTTGTCCCCAAGGTAAAACG				
	Nested real-time PCR *Pm*	Pm_RTPCR_F	ACAGGTGCATCACTTGTATTTTTTC				75.4-76.4°C
		Pm_RTPCR_R	TGCTGGAATTGAAGATAATAAATTAGTAATAACT				
	Nested Real-time PCR *Po*	Po_RTPCR_F	GTTATATGGTTATGTGGAGGATATACTGTT				73.2-74.2°C
		Po_RTPCR_R	CGAATGGAAGAATAAAATGTAGTACG				

*35 cycles when nested real-time PCR, 45 cycles when real-time PCR alone.

### Quality assurance

For each real-time PCR run, negative and positive “in house” prepared controls were used to validate both DNA extraction and real-time PCR runs.

For the validation of the DNA extraction, a set of six control blood spot confetti was added in the 96-well plate prior to the DNA extraction steps (two negative controls and four positive controls: two HPC and two LPC). The lowest concentrations are close to the detection limit of the test, to be sure that the detection limit remains the same over the different tests.

For the real-time PCR screening runs, a set of four controls (two DNA positive controls; a HPC and a LPC, and two negatives; water) were added in the 96-well plate prior to performing the real-time PCR run. Each lot of “in house” prepared quality controls were tested and validated at the Institut Pasteur du Cambodge (IPC) before being used in the field studies. For the validation of the real-time PCR species runs, the plasmid constructs previously described were used in each PCR assays. Details are provided in Table 2.

**Table 2:** **List of the controls used in each PCR assays for the validation of DNA extraction and**/**or the real**-**time PCR runs**

**QC name**		**Number used in 96-well plates**	**Type**	**Concentration**
PCR HPC	PCR High Positive Control	1	Pf 3D7 DNA extract (QiaAmp DNA mini kit)	0.1 ng/μl
PCR LPC	PCR Low Positive Control	1	Pf 3D7 DNA Extract (QiaAmp DNA mini kit)	0.001 ng/μl
PCR NC	PCR Negative Control	2	H2O	N/A
Ext HPC	Extraction High Positive Control	2	Dried blood spot (4mmØ ) with Pf 3D7 blood	500 parasites/μl
Ext LPC	Extraction Low Positive Control	2	Dried blood spot (4mmØ ) with Pf 3D7 blood	5 parasites/μl
Ext NC	Extraction Negative control	2	Dried blood spot (4mmØ ) with Pf negative blood	No parasite
*Pf*	*P. falciparum* Positive Control	1	Plasmid (*Pf* 400 bp insert)	0.001 ng/μl
*Pv*	*P. vivax* Positive Control	1	Plasmid (*Pv* 400 bp insert)	0.001 ng/μl
*Pm*	*P. malariae* Positive Control	1	Plasmid (*Pm* 400 bp insert)	0.001 ng/μl
*Po*	*P. ovale* Positive Control	1	Plasmid (*Po* 400 bo insert)	0.001 ng/μl

For all PCR controls, C_t_ values were plotted on an x-chart to monitor assay precision deviation over time. Additionally, all reagent lots (primers, master mix, saponin solution and Instagene® matrix) were validated using controls at IPC before departure on the field and then again in the mobile laboratory prior to the start of each survey.

### Ethical considerations

The entire study protocol of MalaResT project was approved by three independent ethical committees: The Institutional Review Board of Institute for Tropical Medicine Antwerp, The Ethics Committee of the University Hospital of Antwerp and the National Ethics Committee for Health Research in Cambodia.

### Field study: moving PCR detection in the field

#### Study design and objective

The malaria PCR detection method described in this paper was applied in the context of the two-year study “Repellents as added control measure to long lasting insecticidals nets to target the residual transmission in Southeast Asia: a step forwards to malaria elimination” (NCT01663831). The purpose of this study was to raise evidence on the effectiveness of mass use of topical repellents in addition to long-lasting insecticide nets (LLINs) in controlling malaria infections. The epidemiological efficacy of repellents was evaluated in Rattanakiri Province, eastern Cambodia, on the prevalence of malaria parasite carriers detected by PCR. To achieve this goal, 98 communities consisting of one or more neighbouring villages were randomly assigned to one of two treatment arms (LLINs and LLINs + repellent). Four cross-sectional surveys were planned over a two-year period, one at the start and one at the end of the malaria season. During each survey the aim was to collect blood samples of 65 randomly selected participants within each community. Results presented here are from the survey performed in the mobile laboratory over a 20-day period in October-November 2012.

#### Data flow

Upon reception of samples in the mobile laboratory, sample identifications were entered twice, by two technicians, in a “real-time PCR worksheet”. After PCR run completion, PCR runs and worksheets were sent by email to IPC for validation. Results were double-checked and positive sample identifications were sent back to the field team by email (Figure 2, Panel B).

## Results

### Malaria PCR detection

#### Real-time PCR screening

**Figure 3:**
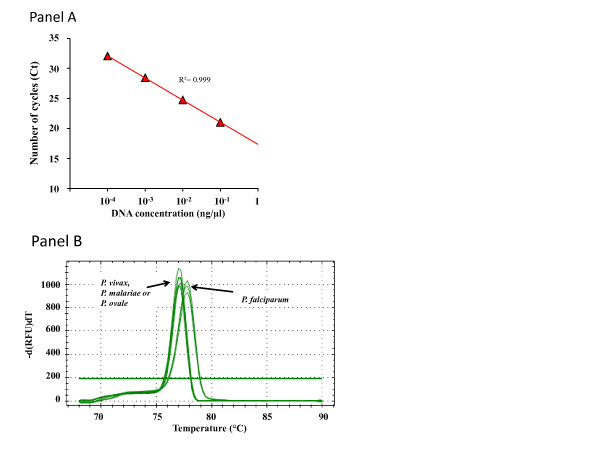
**Linear regression analysis and reproducibility of the real-time PCR screening (Panel A) and melt temperatures (Tm) observed for each *Plasmodium *** **species (Panel B)** ***.***

The threshold of detection was evaluated on *P. falciparum* DNA ten-fold dilutions. Linear regression analysis indicated that the PCR assay has a reproducible linearity over 10^-4^-fold range (R^2^ > 0.999). The efficiency of amplification was higher when an annealing temperature of 58°C was used instead of 60°C (92 and 87%, respectively). However, the annealing temperature of 60°C was preferred as it allowed a reduction of the background noise, facilitating results interpretation, without affecting the threshold of detection of 10^-4^ ng/μl (Figure 3, Panel A).

To compare the performance of the real-time PCR screening and the real-time PCR species, the screening assay was also tested on ten-fold serial dilutions of each species-specific plasmids (*P. falciparum*, *P. vivax*, *P. malariae*, *P. ovale*.), and was capable of detecting *P. falciparum*, *P. malariae*, *P. ovale* plasmid DNA down to 10^-8^ ng/μl, and *P. vivax* plasmid DNA down to 10^-7^ ng/μl.

Melting temperatures (Tm) were consistently higher on *P. falciparum* amplicons (Tm ~78°C) compared to amplicons of other *Plasmodium* species (Tm ~77°C for *P. vivax* and *Plasmodium knowlesi*, Tm ~76.8°C for *P. malariae* and *P. ovale*). The Tm ranging from 76.4 to 78.4°C was considered specific for *Plasmodium* (Figure 3, Panel B). No amplification was observed using *Plasmodium* negative blood (human DNA at 0.1 ng/μl).

The detection threshold of the entire method (including sample volume analysed, DNA extraction and real-time PCR screening) was assessed on a set of serial dilutions of *P. falciparum* blood (1,000 to 0.01 parasites/μl) spotted on filter paper. Eight replicates per dilution were tested twice (except dilution 5 and 2 parasites/μl tested once). On both assays, the real-time PCR screening was capable of detecting parasite DNA in 100% of samples having a parasitaemia at 1,000, 100, 10, 5 and 2 parasites/μl. At 1 parasite/μl, 13/16 (81%) samples were positive (7/8 in assay 1 and 6/8 in assay 2, respectively). No amplification curves were observed for samples at 0.1 parasite/μl or less. The last consistently positive dilution corresponds to 2 parasites/μl whole blood (~10 parasites on the blood spot) (Table 3).

**Table 3:** **Threshold of detection of the real**-**time PCR screening** (**including sample volume analysed**, **DNA extraction and real**-**time PCR screening**): **percentage of positive results according to the dilution of parasites**

**Dilution (parasites/μl)**	**% of positive results (No positives/No tested)**		
	**Assay 1**	**Assay 2**	**Average**
1,000	100% (8/8)	100% (8/8)	100% (16/16)
100	100% (8/8)	100% (8/8)	100% (16/16)
10	100% (8/8)	100% (8/8)	100% (16/16)
5	ND	100% (8/8)	100% (8/8)
2	ND	100% (8/8)	100% (8/8)
1	87% (7/8)	75% (6/8)	81% (13/16)
0.1	0% (0/8)	0% (0/8)	0% (0/8)
0.01	0% (0/8)	ND	0% (0/8)

#### Real-time PCR species

*Plasmodium* species identity for each species-specific plasmid was confirmed by sequencing (*P. ovale* plasmid corresponds to the sub-species *P. o. wallikeri*).

#### Sensitivity

*Plasmodium falciparum* and *P. vivax* real-time PCR species assays were capable of detecting plasmid DNA down to 10^-7^ ng/μl, while *P. malariae* and *P. ovale* real-time PCR species assays were 100% positive down to 10^-8^ ng/μl.

#### Specificity

The four real-time PCR species assays showed specific amplification when tested on all different plasmids (0.01 ng/μl), except with *P. malariae* plasmid, which showed a low amplification curve in *P. vivax* real-time PCR reaction. However, no unspecific amplifications were observed with higher dilution of *P. malariae* plasmid.

Primers targeting *P. knowlesi* were originally designed, but removed from the assay because they were not specific.

When using artificial mixed infections (various concentrations of “targeted plasmid” with constant concentration at 0.01 ng/μl of “added plasmid”), the performances of the real-time PCR species were not affected, even when the “added” plasmid was 100,000 times more concentrated than the “targeted plasmid” (Additional file 3).

#### Nested real-time PCR species

In order to save DNA templates and to increase the sensitivity, a nested PCR step was added using genus-specific primers prior to the real-time PCR species. With this additional step, only 5 μl of DNA extract was enough to perform the four real-time PCR species (instead of 20 μl). This format was evaluated on the same dilutions of plasmids, and showed similar or higher sensitivity than the real-time PCR species assays.

### Comparison with the *18S rRNA* nested PCR

#### Real-time screening PCR versus genus-specific 18S rRNA nested PCR

The real-time PCR screening assay was compared to the *18S rRNA* nested PCR on a set of 175 African DNA samples, previously screened on-site (Uganda and Burkina Faso) with the nested *18S rRNA* PCR. Samples were first retested with the genus-specific primers of the *18S rRNA* nested PCR at IPC and then with the real-time PCR screening assay. Three samples showed discordant results between the two nested *18S rRNA* PCR runs and were removed from the panel. Among the remaining 172 samples, 23% (39/172) were found positive with the *18S rRNA* nested PCR and 31% (54/172) with the real-time PCR screening (Table 4, Panel A). The 15 discordant samples had the highest C_t_ values among the 54 samples detected as positives by the real-time screening PCR (concordant samples, mean C_t_ = 25 cycles and discordant samples, mean C_t_ = 35 cycles); those with a C_t_ value >35 cycles were systematically negative with the *18S rRNA* nested PCR, while 50% of those with a C_t_ value between 30–35 cycles were found negative with the *18S rRNA* nested PCR (Table 5, Panel B).

**Table 4:** **Performances of the real**-**time screening PCR and the genus**-**specific *18S rRNA *** **nested PCR: concordant and discordant data**

**PCR detection**		**Real-time screening PCR**		
		**Positive**	**Negative**	**Total**
Genus-specific *18S rRNA* nested PCR	Positive	39	0	39
	Negative	15	118	133
	Total	54	118	172

**Table 5:** Performances of the real-time screening PCR and the genus-specific 18S rRNA nested PCR: according to Ct range

**Real-time screening PCR Ct range**	**Number of positive samples by using**	
	**Real-time screening PCR**	**Genus-specific** ***18S rRNA*** **nested PCR (% of concordance)**
16 to 29 cycles	34	34 (100%)
30 to 35 cycles	10	5 (50%)
> 35 cycles	10	0 (0%)

#### Nested real-time PCR species assay versus species-specific 18S rRNA nested PCR

The results of the 54 positive samples tested with the real-time PCR species assay and the species-specific *18S rRNA* nested PCR are presented in Table 6 (Panel C). Using both PCR, single *P. falciparum* or *P. ovale* infections were detected, while *P. malariae* was found only in mixed infection (with *P. falciparum* or *P. ovale* or both) and *P. vivax* was not detected.

**Table 6:** Performances of the real-time screening PCR and the genus-specific 18S rRNA nested PCR: species identification

**Results**	**Malaria PCR detection**		
	**Species-specific** ***18S rRNA*** **nested PCR**	**Nested real-time PCR species**	
		**Concordant samples**	**Discordant samples**
*P. falciparum*	31	29	9
*P. ovale*	2	2	2
*P. falciparum*/*P. ovale*	3	3	1
*P. falciparum*/*P. malariae*	2	4	0
*P. falciparum*/*P. ovale*/*P. malariae*	1	1	0
No species	0	0	3
Total	39	39	15

Among the 39 samples found positive with both genus-specific PCR, 35 (90%) showed concordant species results. For the four discordant samples: three samples were detected as single *P. falciparum* with the species-specific *18S rRNA* nested PCR but as mixed infection with the nested real-time PCR species (*P. falciparum*/*P. malariae*, N = 2 and *P. falciparum*/*P. ovale*, N = 1) and one sample was detected as *P. falciparum* with the nested real-time PCR species but as mixed infection with the species-specific *18S rRNA* nested PCR (*P. falciparum*/*P. ovale*). Among the 15 samples missed by the *18S rRNA* nested PCR, 75% were classified as single *P. falciparum*. The nested real-time PCR species failed to amplify three samples detected as positive using real-time screening PCR (“*P. falciparum*-like Tm” (78–78.2) and Ct >36). The most likely hypothesis is that these samples were low positives for *P. falciparum*, below the limit of detection of the *P. falciparum*-specific real-time PCR assay (the limit of detection of the real-time screening assay appears to be better for *P. falciparum*).

### Implementation of malaria PCR detection into the field: study results

The set-up described here was successfully implemented in the field. From 23 October to 15 November, 2012, a total of 4,999 blood samples were collected and screened for malaria parasites in the mobile laboratory in Rattanakiri Province, in northeast Cambodia. An average of 240 clinical samples (+40 quality control samples) was tested every day, six/seven days per week; 97.7% of the results were available in less than 24 hours after sample collection and malaria-positive individuals were treated in less than 48 hours after the collection. Only 2.3% of the results (117/4,999) were delayed due to invalid runs (which were repeated the next morning), and were delivered in less than two working days after the collection.

### Prevalence of the malaria species

Among the 4,999 collected samples, 244 samples (4.9%) were detected positive for malaria and analysed for malaria species identification. All the four human *Plasmodium* species were detected. In general, *P. vivax* was present in 61.1% of the positive samples, *P. falciparum* in 45.9%, *P. malariae* in 7.0%, and *P. ovale* in 2.0%.

Single malaria infections were found in 86.9%. *Plasmodium vivax* was the most prevalent single infection (49.2%) followed by *P. falciparum* (34.0%), *P. malariae* (3.3%) and *P. ovale* (0.4%). Mixed infections were also frequent (13.1%) associating two species (*P. falciparum*/*P. vivax*, 8.6%; *P. falciparum*/*P. malariae*, 0.8%; *P. falciparum*/*P. ovale*, 0.4%; *P. vivax*/*P. malariae*, 0.4%; *P. vivax*/*P. ovale*, 0.4%), three species (*P. falciparum*/*P. vivax*/*P. malariae*, 1.6%; *P. vivax*/*P. malariae*/*P. ovale*, 0.4%) or four species (0.4%).

### Quality control data

Run validation was based on the results of ten internal quality controls analysed per run (one PCR HPC, one PCR LPC, two PCR NC, two Ext HPC, two Ext LPC, two Ext NC, see Table 2). A total of 830 quality control samples were analysed, representing 16% of the PCR tests performed. As presented in Additional file 4, no false negative and six false positives were observed: four positives out of 166 PCR negative controls and two positives out of 166 extraction negative controls (the whole plate was retested in these cases).

The C_t_ values of the 166 PCR controls were plotted on a x-chart to follow assay precision over time and only one PCR HPC and one PCR LPC were observed to be out of the two standard deviation (SD) limit (Additional file 5). The precision of the real-time PCR assay was also assessed by the coefficient of variation (CV) and SD of the replicate C_t_ measurements (n = 83 x2) for the two PCR positive controls. The CV for the mean C_t_ values obtained with the PCR HPC (mean C_t_ value = 21.07) and PCR LPC (mean C_t_ value = 29.84) was 3.1 and 2.4%, respectively, demonstrating a very low inter-assay variability (CV <5%). The average melt Tm values were 78.08°C (SD = 0.15 and CV% = 0.20) and 78.05°C (SD = 0.13 and CV% = 0.16) for the HPC and the LPC, respectively.

## Discussion

In the context of malaria elimination, the development of new diagnostic approach capable to detect low infection for mass screening in field settings is essential [14]. In this study, a reliable and cost-effective molecular assay for malaria diagnostic was adapted for high throughput testing and transferred into a mobile laboratory.

The real-time PCR screening assay showed good amplification efficiency and was capable to detect *Plasmodium* 3D7 DNA as low as 10^-4^ ng/μl. When combined together with the Instagen DNA extraction, the detection limit using 5 μl of blood on dried blood spot was 2 parasites/μl. The melt curve analysis allowed the differentiation of falciparum from non-falciparum malaria positive samples (melt temperatures were consistently higher for falciparum amplicons compared to other species). The real-time PCR screening assay was also found more sensitive than the reference *18S rRNA* nested PCR, by detecting more often very low parasitaemia. This increased sensitivity is likely related to the targeted gene and the method used to detect the PCR products. Indeed, in malaria parasites, the mitochondrial genome is presented at a higher copy number than *18S rRNA* gene, and, while early ring-stage parasite typically have one mitochondrial organelle, mature gametocytes have many. Moreover, the melt curve analysis step following the real-time PCR allowed the differentiation of specific/unspecific late amplification curves (generally occurring at C_t_ > 35), and thus, enabled an increase in the number of amplification cycles up to 45.

Species identification of positive samples was highly comparable between the two PCR methods used, with 90% concordant results. The discordances observed in four/39 samples were due to a failure to amplify the species present at very low level in a mixed infection by the *18S RNA* nested PCR in three cases, and by the real-time in one case. This finding suggests that the real-time PCR species assays have an improved ability to detect minor species in mixed infection. Indeed, plasmid mixing experiments showed that the presence of a highly concentrated species did not impact the detection of the minor species, even with a ratio of 1:100,000.

As Cambodia is a low transmission area, the adopted strategy was to first screen for malaria positive cases using a highly sensitive real-time PCR, followed by a species identification of those positive samples. This approach offers the advantages of reducing result turnaround times and PCR costs. A first round of amplification with genus-specific primers can be optionally added prior to performing the four real-time PCR species assays. Although the real-time species assays alone demonstrated good performances when tested on quality controls (similar to the real-time screening assay), the addition of an outer PCR was observed to be beneficial when working with clinical samples; likely because DNA extracted by Instagen method is not very stable and sensitive to freeze/thaw cycles, and so, low quantities of parasites DNA may start being degraded during the storage time between the “screening test” and the “species test”. This additional step also offers the advantage of reducing the quantity of DNA templates needed to perform the analysis.

The next challenge after the validation of the methodology was to transfer it from the laboratory to the mobile laboratory. In less than one month, a total of 4,999 samples were screened for malaria parasites in the province of Ratanakiri in October-November 2012. Five four-person teams conducting sample collections and two laboratory technicians were permanently present in the field. The technicians typically performed four DNA extractions (96-well plate format) and four real-time PCR runs per working day. An average of 240 clinical samples and 40 quality controls were analysed per day. More than 97% of the results were delivered in less than 24 hours, allowing study participants identified as malaria positive and lacking any symptoms, to be offered a treatment free of charge in less than 48 hours.

Quality control performances demonstrated the real-time PCR screening to have a very good sensitivity and specificity (100% of both extraction and PCR positive controls being positive, even for the lowest positive control at 5 parasites/μl and 98.2% of negative controls being negative). In addition, a satisfactory low interassay coefficients of variation (CV <5%) proves its reproducibility.

As the goal was to develop a malaria diagnostic intended for large-scale malaria screening studies, the challenge was to keep the testing price at a minimum. The average cost for the real-time PCR screening, including DNA extraction, is estimated at 2.75 USD per sample, whereas the identification of the four *Plasmodium* species was performed at 3.75 USD per sample. In addition, the global cost of the mobile laboratory fully equipped for PCR was around 200,000 USD.

The main limitations of the PCR developed are related to the DNA extraction step and, mainly to the volume of blood analysed. Indeed, for low parasitaemia samples, it is obvious that the higher the volume of blood collected, the higher the probability to pick up parasites. When collecting 5 μl of blood, the absolute limit of detection that can be achieved is one parasite/5 μl blood (0.2 parasite/μl) if the entire DNA extract is analysed. In the protocol described here, only one/ten of the DNA extract is analysed, raising the theoretical detection limit to 2 parasites/μl blood. When the overall diagnostic method was assessed on a serial dilution of falciparum parasites, the last consistently positive dilution was actually 2 parasites/μl. Increasing the amount of blood tested during mass screening studies is technically challenging as blood samples are usually collected from finger prick, and high throughput testing is facilitated by 96-well plate format collection. Moreover, the use of a higher volume of blood would raise another issue: the high quantities of human DNA that will interfere with parasite DNA amplification by PCR. To date, no (or little) information is available on the real distribution of asymptomatic parasite carriers and the minimum parasitaemia in malaria parasite carriers, therefore, the proportion of infection being missed by a screening method with a detection threshold of 2 parasites/μl is impossible to predict.

Another limitation was related to the specificity of the real-time PCR species; indeed, samples highly positive for *P. malariae* could be misclassified as very weak positive for *P. vivax*. Thus, special attention must be paid if one sample simultaneously presents an early amplification curve for *P. malariae* (C_t_ <18) and a late amplification curve for *P. vivax* (C_t_ >35), even if this scenario appears to be rare. Finally, regarding the detection of *P. knowlesi* infection, the primers designed were found to be unspecific and were removed from the assay. As a consequence, a *P. knowlesi* infection would not be missed by the real-time PCR screening, but would be identified as a *P. vivax* infection by the real-time PCR species assays.

## Conclusions

The main goal of this work was to develop a diagnostic approach allowing the analysis of thousands of clinical samples in field conditions under a restricted time window. A quality control system was implemented to ensure quality of diagnostic results and demonstrated the robustness and reliability of the methods, even when working in a limited laboratory space in a remote area. The operational success of this study proved that the molecular testing and treatment is logistically achievable in field settings. This new diagnostic tool will allow the detection of clusters of asymptomatic carriers and will provide epidemiological information that can be directly used for improving control strategies. Faster results will be of great help for staff in the field to track and treat positive cases. The concept of molecular assays performed in a mobile laboratory could be extended to other countries for the detection of malaria asymptomatic cases but also for the detection of other pathogens where sensitive and rapid diagnostic assays are needed, especially in remote areas. Finally, the mobile laboratory is also a tremendous opportunity to perform malaria parasite culture in the field, especially for *P. vivax*, which does not support a long delay between sample collection and culture.

## Competing interests

The authors declare that they have no competing interests.

## Authors’ contributions

MC, DM, LD, VS, SS, ST, SH, CG, KVR, KPG and CMC conceived the study, and participated in its design and coordination. LC, HL, PMN, VD, MC and DM participated in the design and implementation of the mobile laboratory and it equipment. LC, NK, DD, RE, SC, KL, and MK carried out the molecular works. SK, VS, SH, CK, SU, MS, and BT participated to the samples collection. LC, MC and DM wrote the manuscript. All authors read and approved the final manuscript.

## Supplementary Material

Additional file 1 Pictures of the mobile laboratory. Click here for file

Additional file 2 Blood samples collection. Click here for file

Additional file 3 Qualitative assessment of quality controls during the survey. Click here for file

Additional file 4 Performances of the real-time PCR species using artificial mixed infections (various concentrations of “targeted plasmid” with constant concentration at 0.01 ng/μl of “added plasmid”). Click here for file

Additional file 5 Quantitative assessment of PCR quality controls: Ct values of 830 PCR quality controls plotted on a x-chart, Rattanakiri, Cambodia, 2012. Click here for file
