# Supplementary figures and images for: An innovative tool for moving malaria PCR detection of parasite reservoir into the field
Source: Malar J. 2013 Nov 9;12:405. doi: 10.1186/1475-2875-12-405 (PMC3829804; doi:10.1186/1475-2875-12-405)

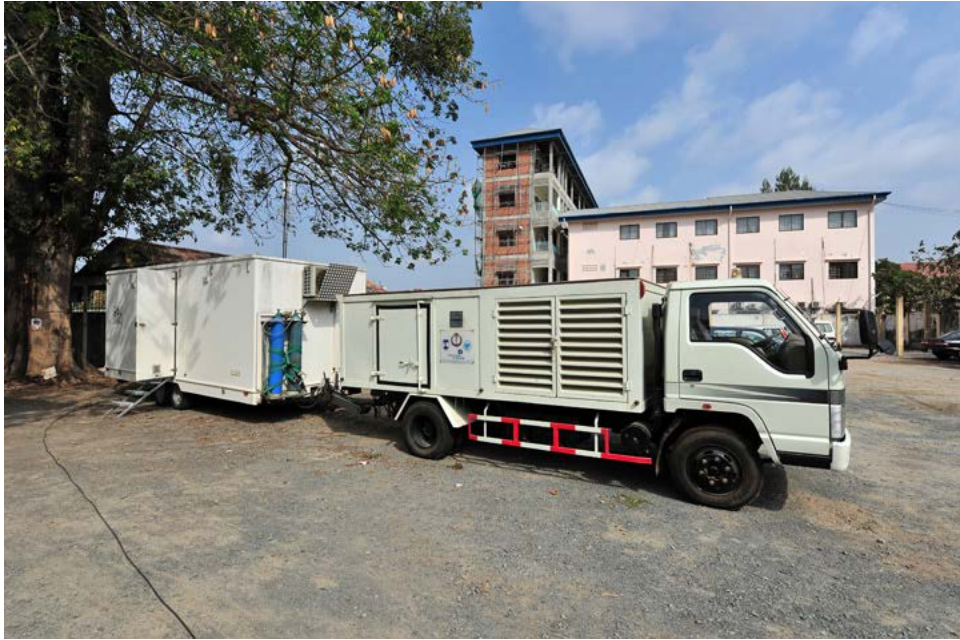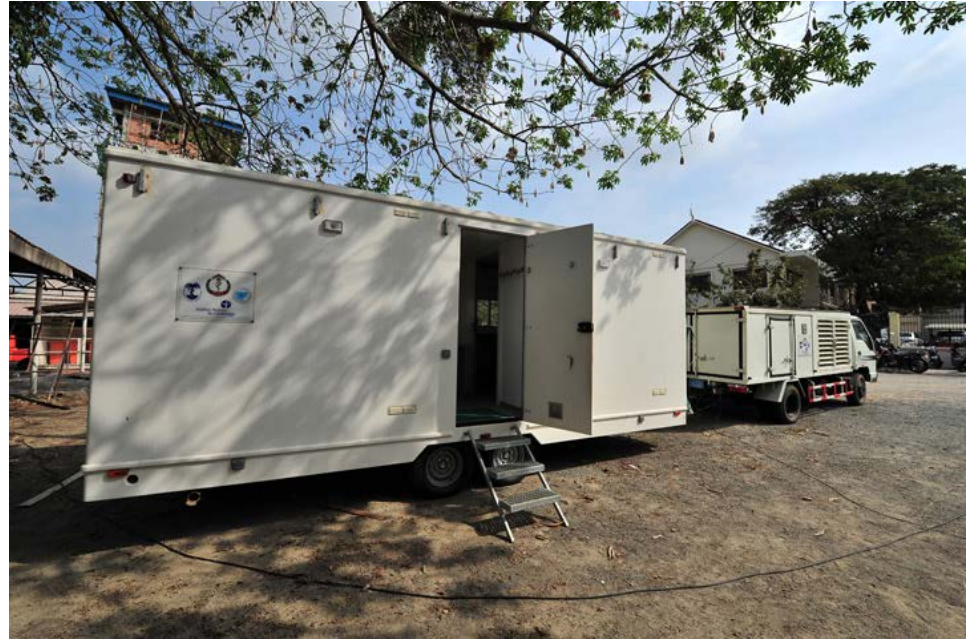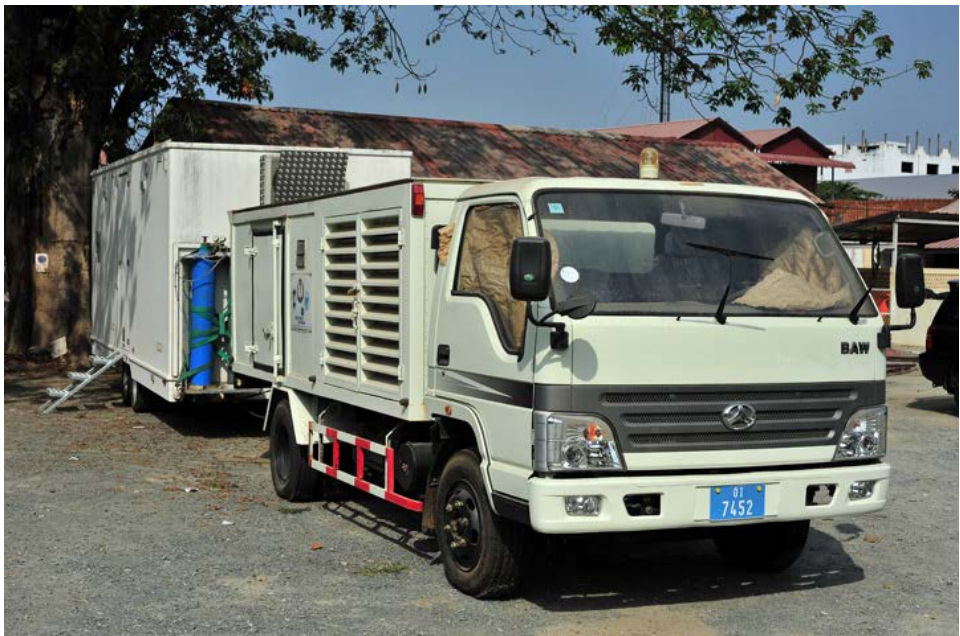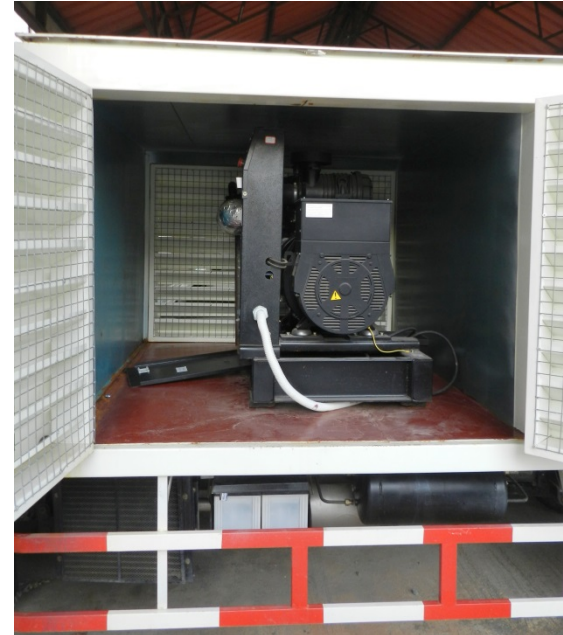

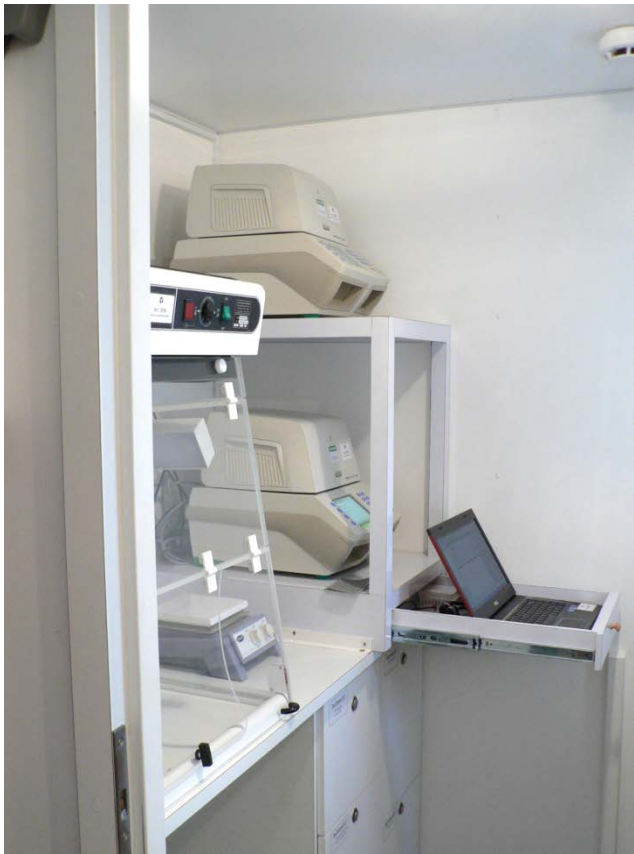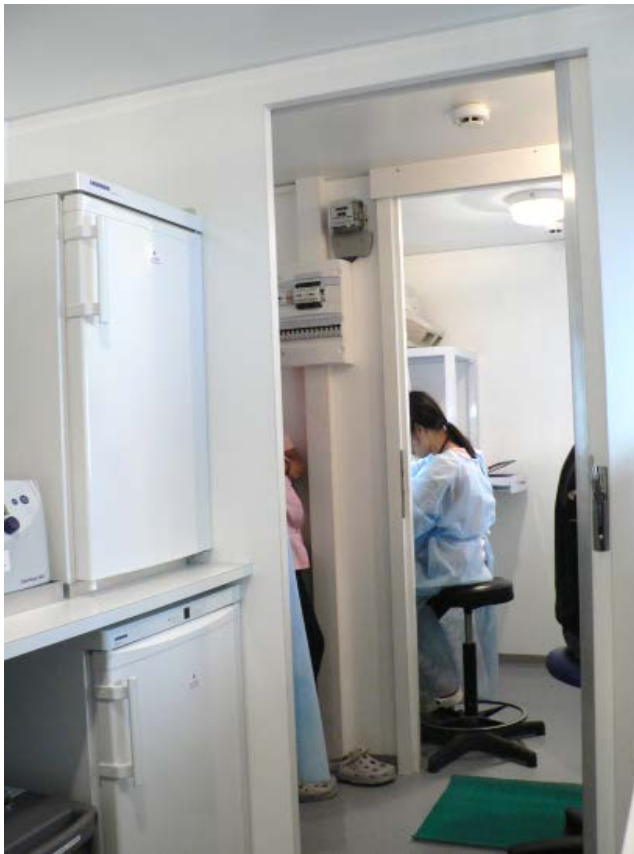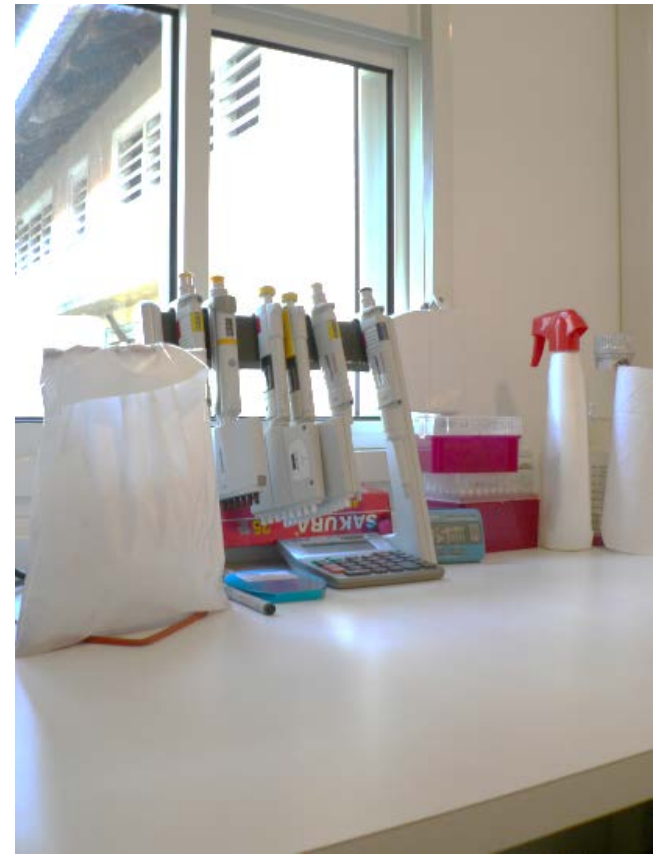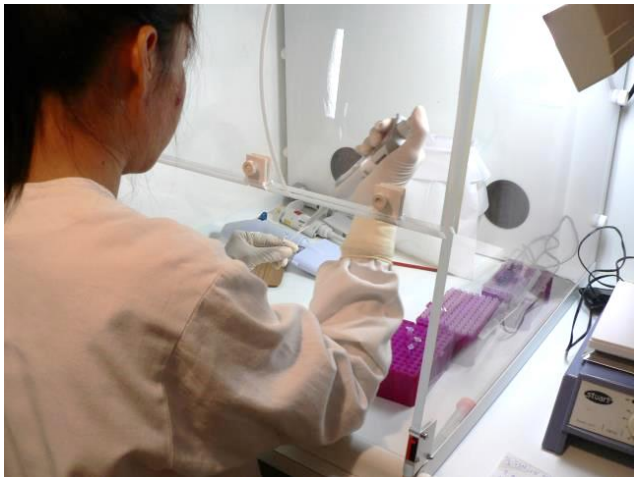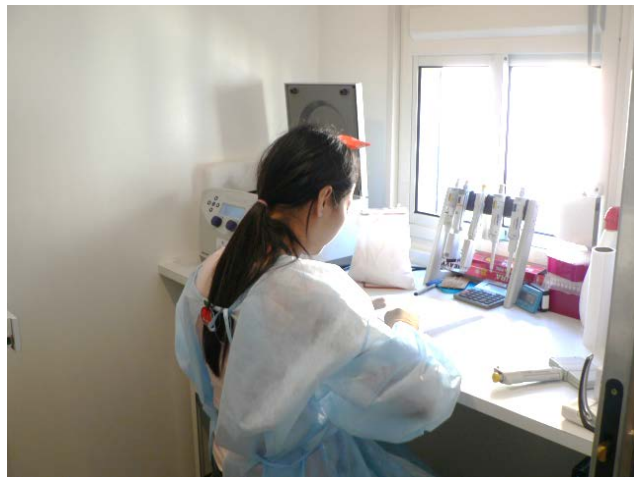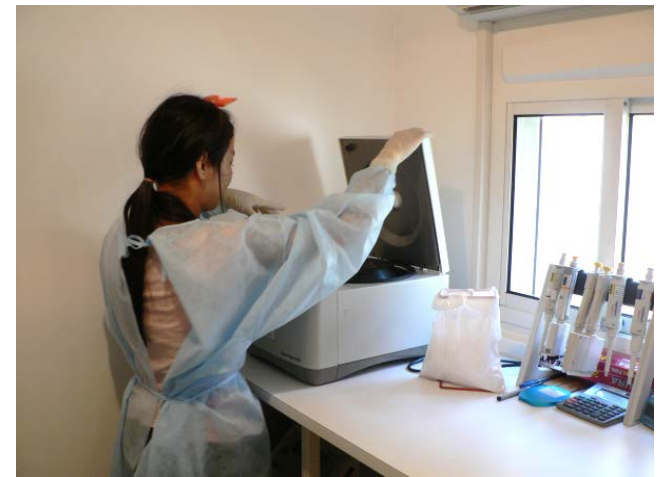

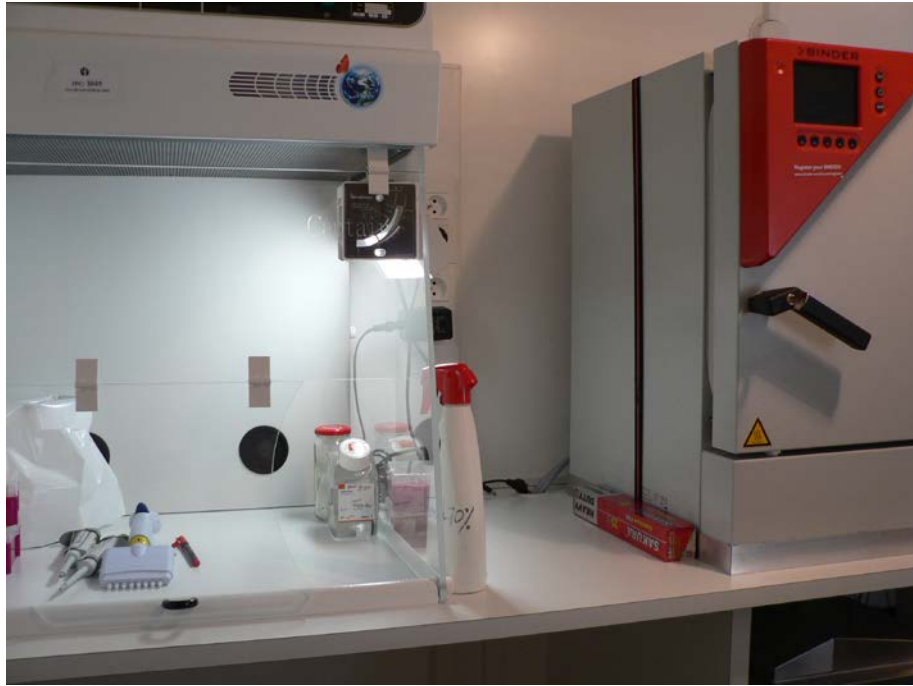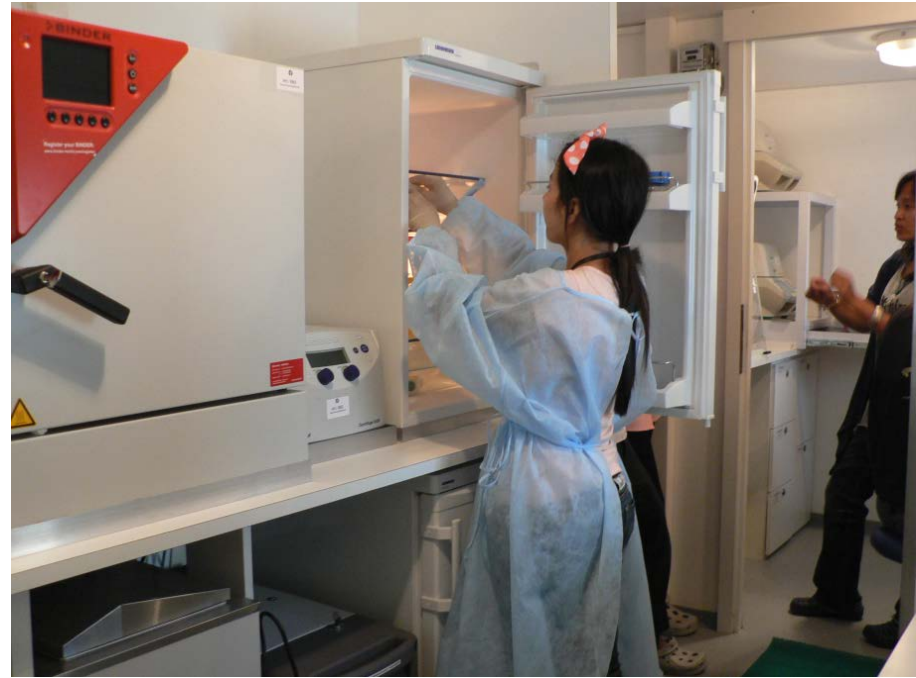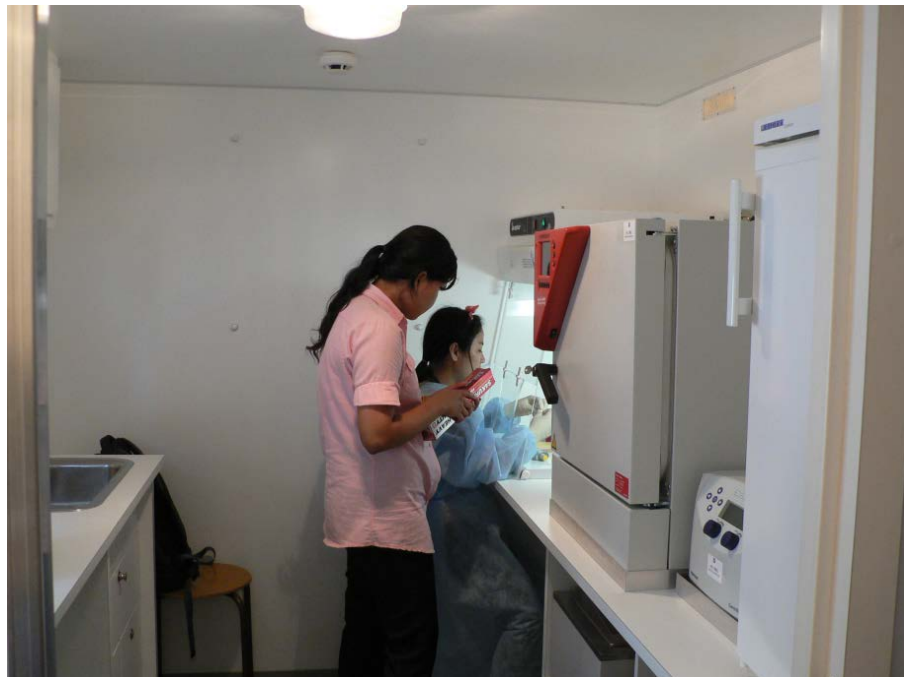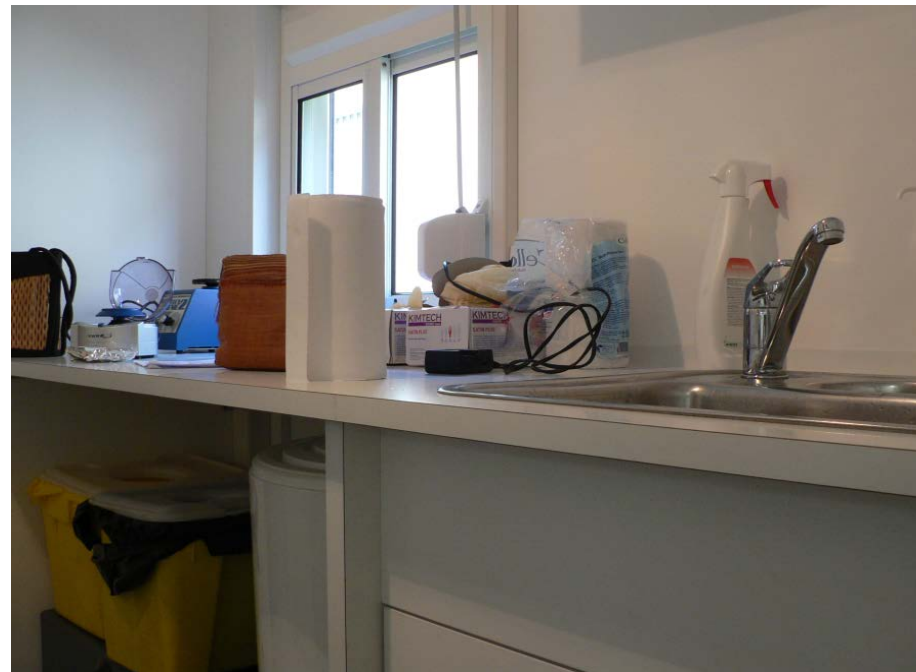

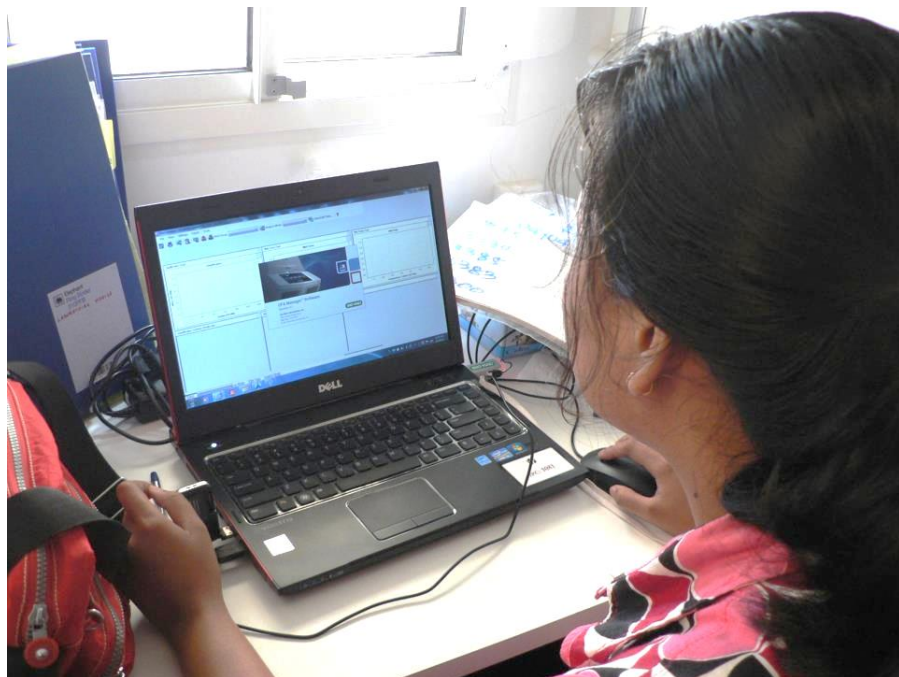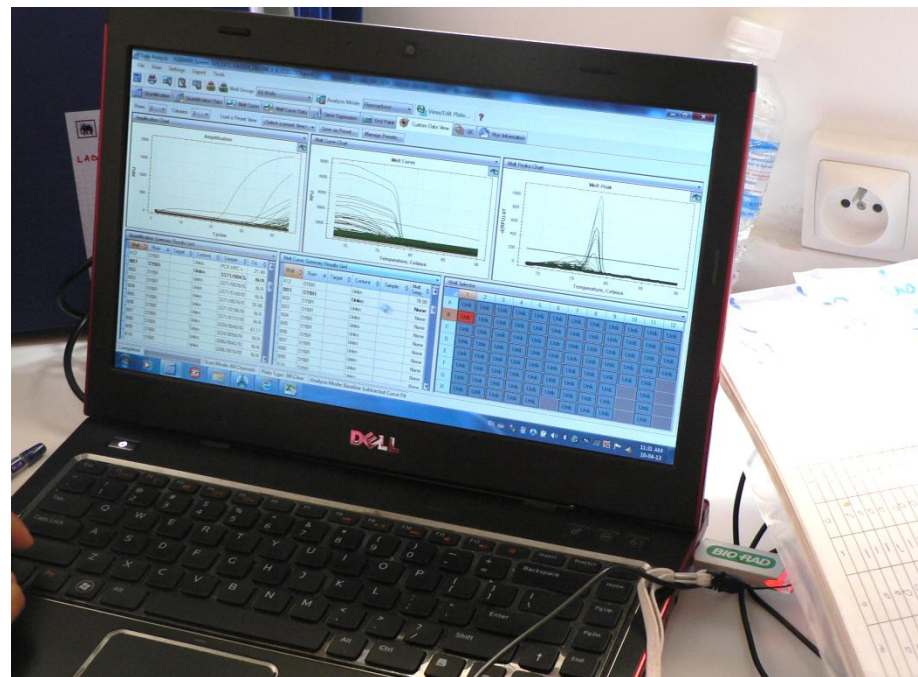

Supplement: Additional file 1 — Pictures of the mobile laboratory. [file 1475-2875-12-405-S1.pdf]

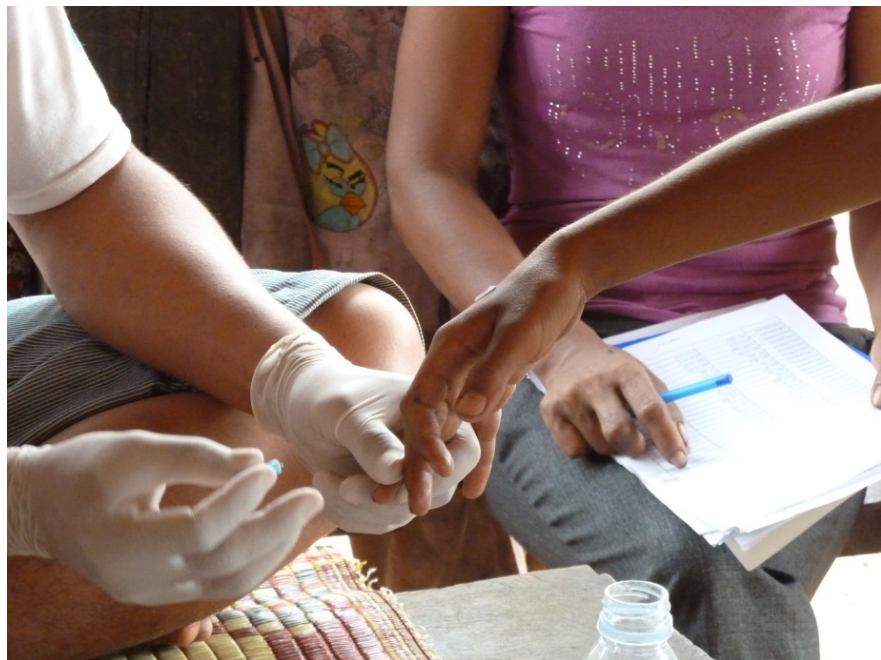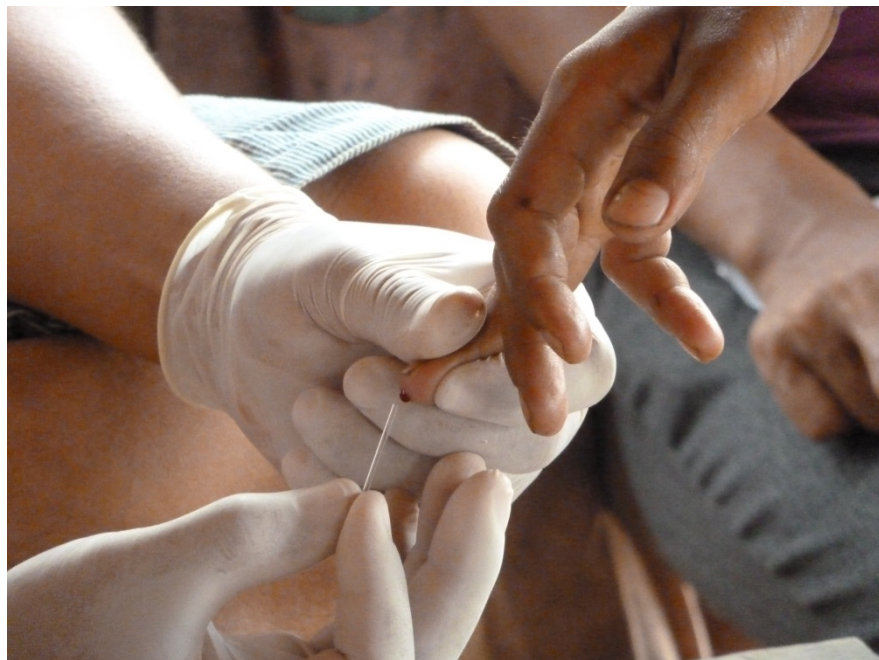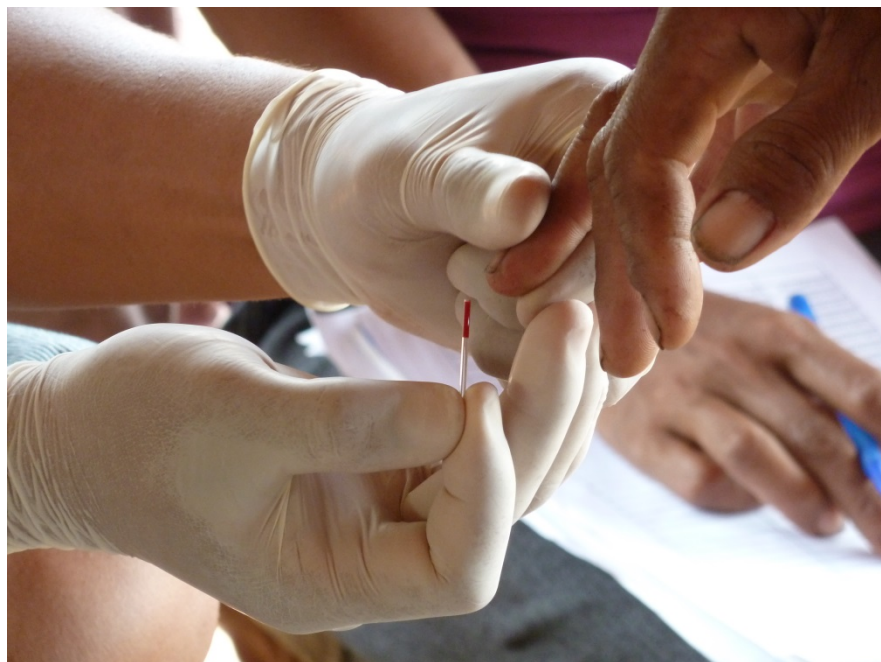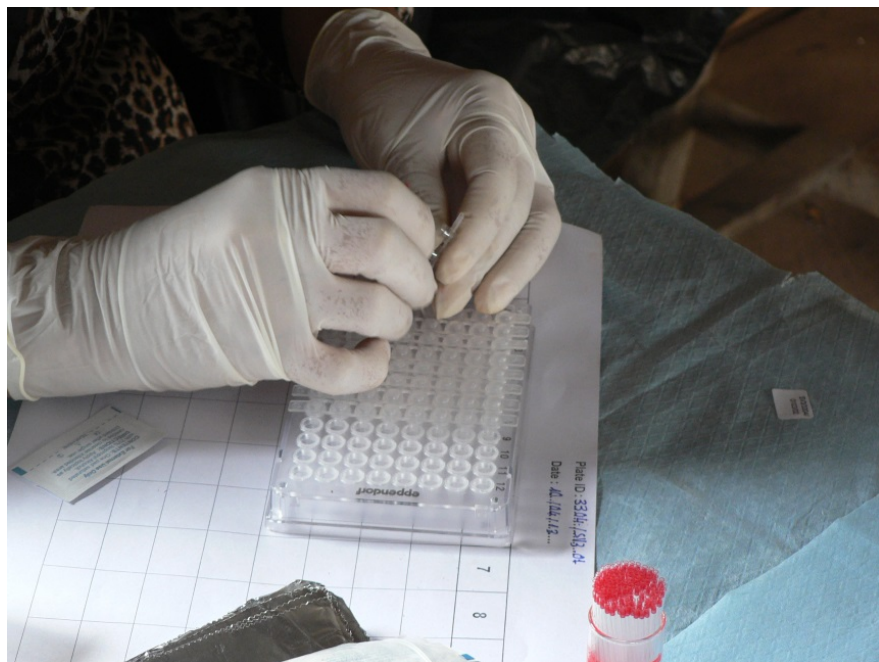

Supplement: Additional file 2 — Blood samples collection. [file 1475-2875-12-405-S2.pdf]

Panel A

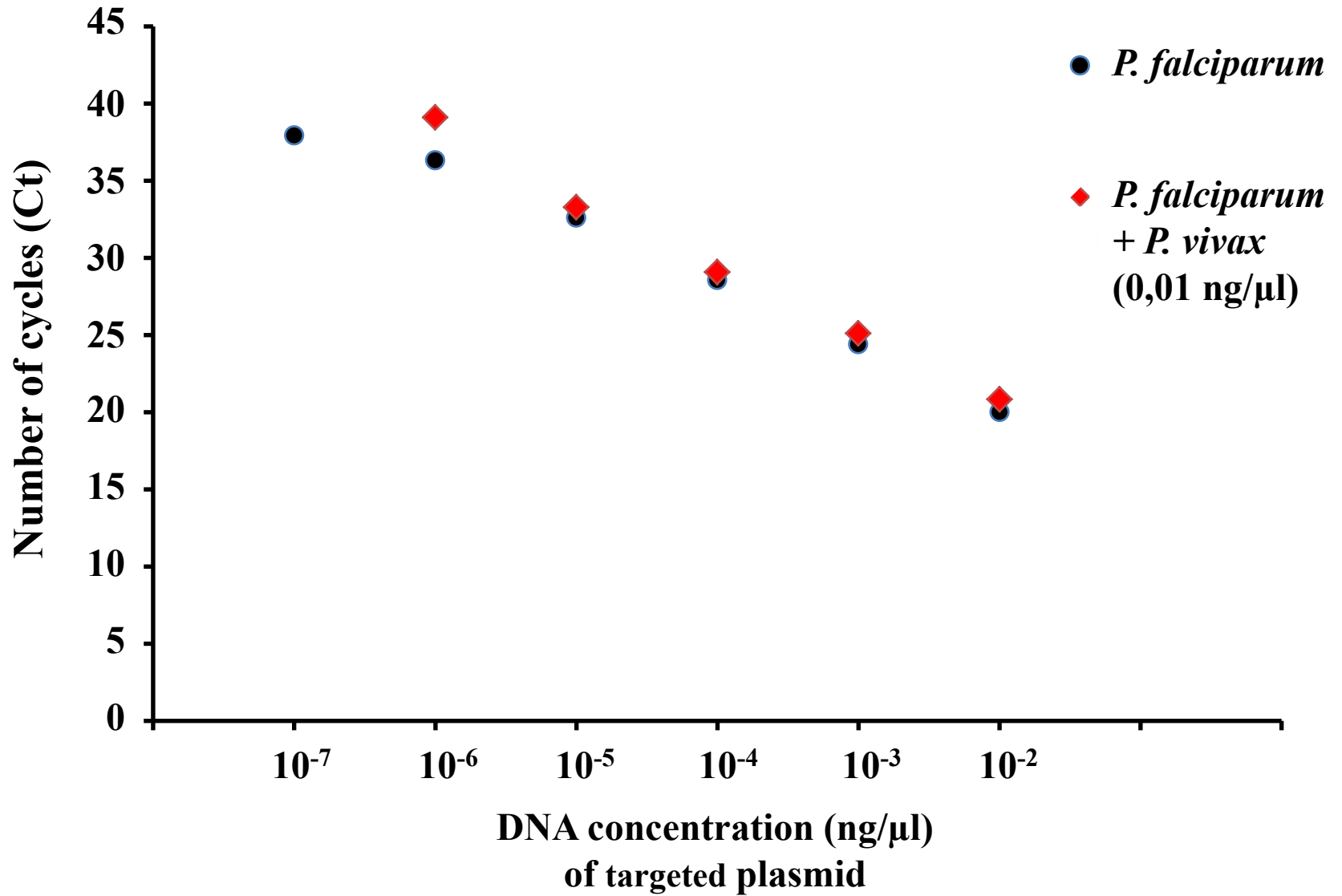

## Panel B

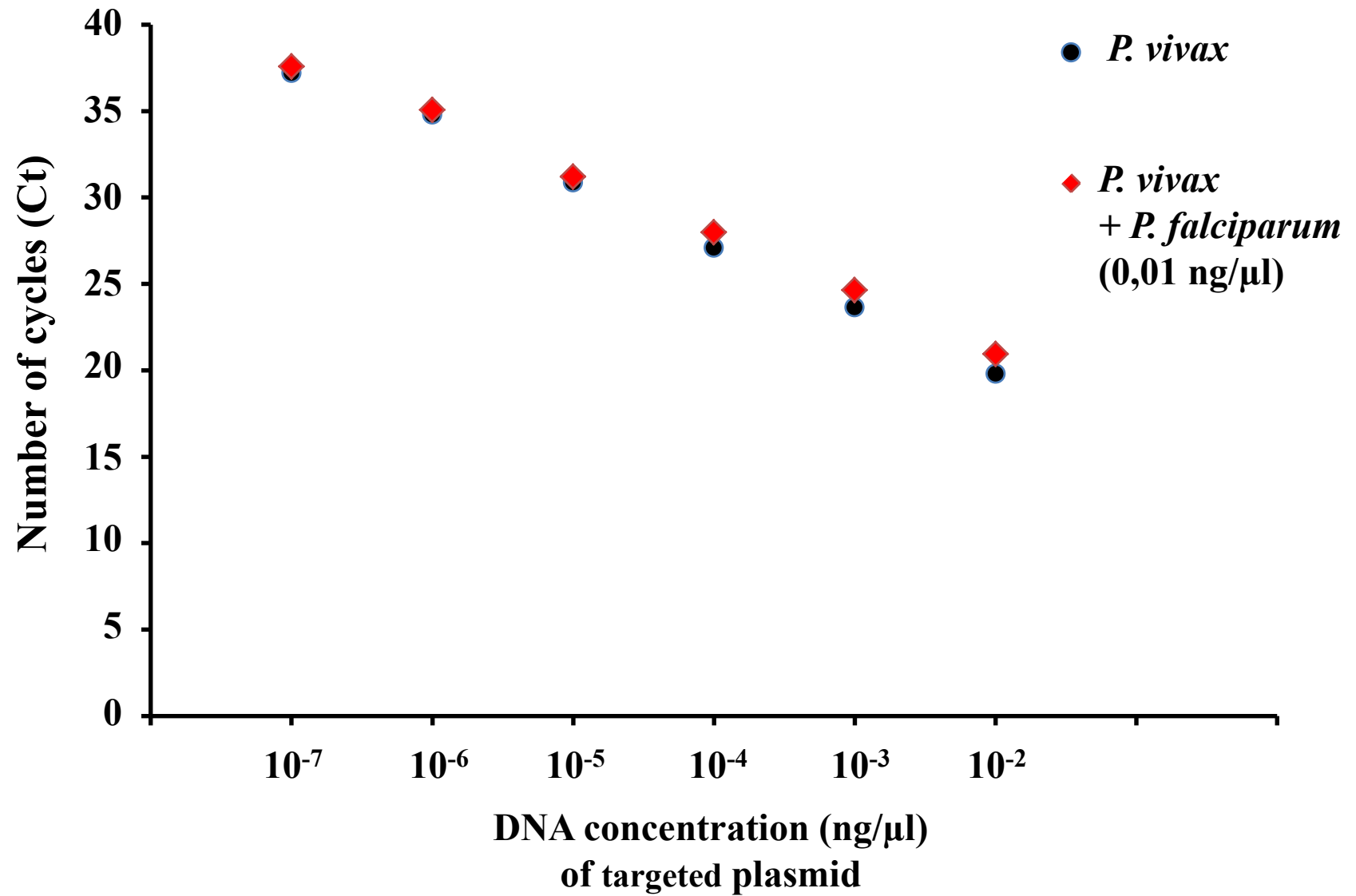

Panel C

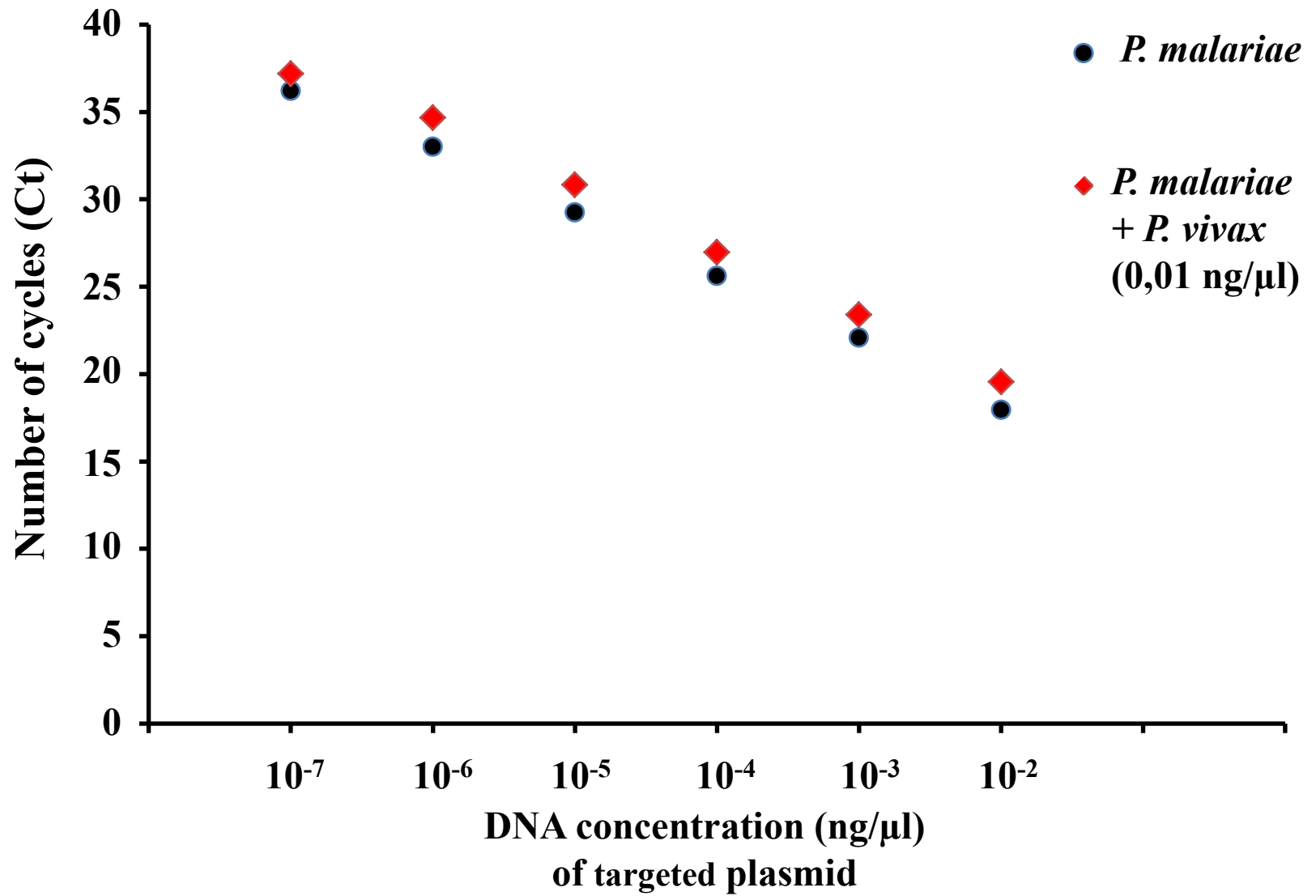

Panel D

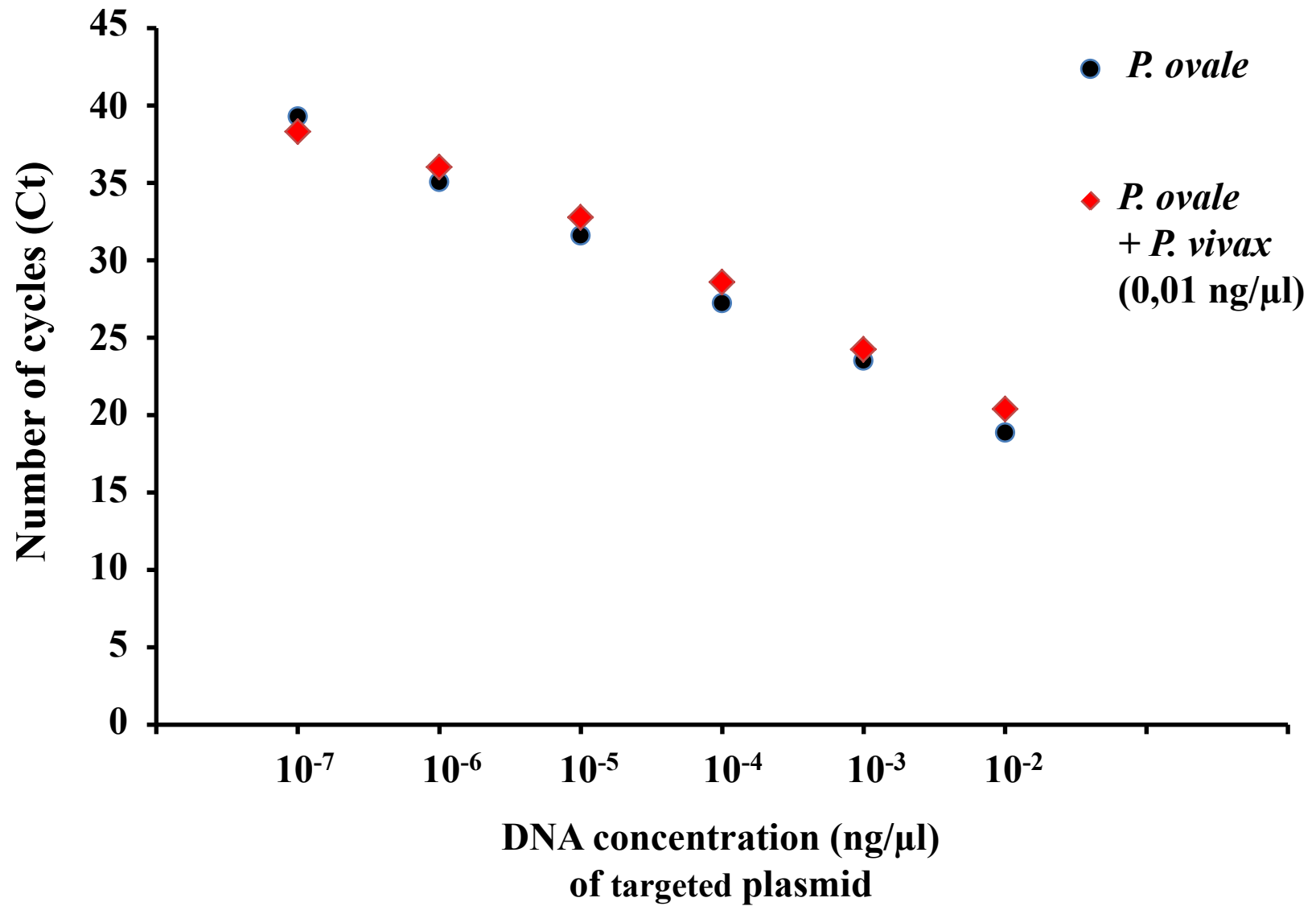

Supplement: Additional file 3 — Qualitative assessment of quality controls during the survey. [file 1475-2875-12-405-S3.pdf]

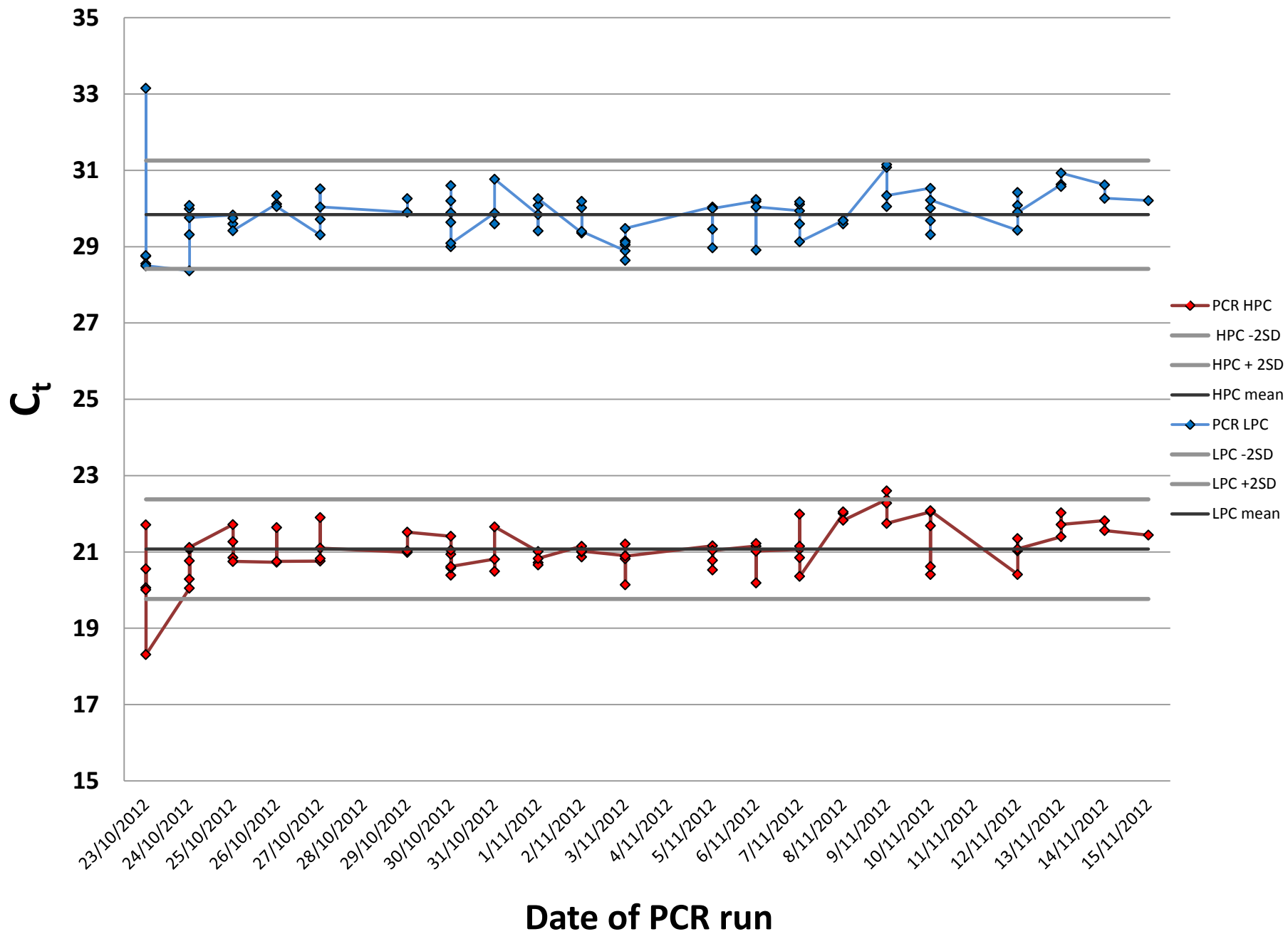

Supplement: Additional file 5 — Quantitative assessment of PCR quality controls: Ct values of 830 PCR quality controls plotted on a x-chart, Rattanakiri, Cambodia, 2012. [file 1475-2875-12-405-S5.pdf]
