# Supplementary material for: An innovative tool for moving malaria PCR detection of parasite reservoir into the field
Source: Malar J. 2013 Nov 9;12:405. doi: 10.1186/1475-2875-12-405 (PMC3829804; doi:10.1186/1475-2875-12-405)
Supplement: Additional file 4 — Performances of the real-time PCR species using artificial mixed infections (various concentrations of “targeted plasmid” with constant concentration at 0.01 ng/μl of “added plasmid”). [file 1475-2875-12-405-S4.pdf]

**Table S3. Qualitative assessment of quality controls during the survey**

| QC sample | Number tested | Number found positive | Number found negative |
|---|---|---|---|
| PCR HPC (3D7 DNA 0.1ng/µl) | 83 | 83 | 0 |
| PCR LPC (3D7 DNA 0.001ng/µl) | 83 | 83 | 0 |
| PCR NC (H2O) | 166 | 4 | 162 |
| Ext HPC (500 parasites/µl WB on DBS) | 166 | 166 | 0 |
| Ext LPC ( 5 parasites/µl WB on DBS) | 166 | 166 | 0 |
| Ext Neg (Negative blood on DBS) | 166 | 2 | 164 |

WB: whole blood; DBS: Dried Blood Spot
